# Ecdysis in a stem-group euarthropod from the early Cambrian of China

**DOI:** 10.1038/s41598-019-41911-w

**Published:** 2019-04-05

**Authors:** Jie Yang, Javier Ortega-Hernández, Harriet B. Drage, Kun-sheng Du, Xi-guang Zhang

**Affiliations:** 1 Key Laboratory for Palaeobiology, Yunnan University, Kunming, 650091 China; 2 MEC International Joint Laboratory for Palaeoenvironment, Yunnan University, Kunming, 650091 China; 3 Museum of Comparative Zoology and Department of Organismic and Evolutionary Biology, Harvard University, 26 Oxford Street, Cambridge, MA 02138 USA; 4 Department of Zoology, University of Cambridge, Downing Street, Cambridge, CB2 3EJ UK; 5 Department of Zoology, University of Oxford, 11a Mansfield Road, Oxford, OX1 3SZ UK; 6 Institute of Earth Sciences, University of Lausanne, Géopolis, CH-1015 Lausanne, Switzerland

## Abstract

Moulting is a fundamental component of the ecdysozoan life cycle, but the fossil record of this strategy is susceptible to preservation biases, making evidence of ecdysis in soft-bodied organisms extremely rare. Here, we report an exceptional specimen of the fuxianhuiid *Alacaris mirabilis* preserved in the act of moulting from the Cambrian (Stage 3) Xiaoshiba Lagerstätte, South China. The specimen displays a flattened and wrinkled head shield, inverted overlap of the trunk tergites over the head shield, and duplication of exoskeletal elements including the posterior body margins and telson. We interpret this fossil as a discarded exoskeleton overlying the carcass of an emerging individual. The moulting behaviour of *A. mirabilis* evokes that of decapods, in which the carapace is separated posteriorly and rotated forward from the body, forming a wide gape for the emerging individual. *A. mirabilis* illuminates the moult strategy of stem-group Euarthropoda, offers the stratigraphically and phylogenetically earliest direct evidence of ecdysis within total-group Euarthropoda, and represents one of the oldest examples of this growth strategy in the evolution of Ecdysozoa.

## Introduction

The process of moulting consists of the periodical shedding (i.e. ecdysis) of the cuticular exoskeleton during growth that defines members of Ecdysozoa^1^, a megadiverse animal group that includes worm-like organisms with radial mouthparts (Priapulida, Loricifera, Nematoida, Kinorhyncha), as well as more familiar forms with clawed paired appendages (Euarthropoda, Tardigrada, Onychophora). Ecdysozoans are among the most ubiquitous group of animals throughout the Phanerozoic, and by virtue of their ecological success they have become a fundamental component of the biosphere since their appearance during the Cambrian Explosion more than 500 million years ago^2,3^. Given their diversity of forms, the phylogenetic relationships between extant Ecdysozoa are usually recognized through similarities at the molecular level^1–4^. Despite the scarcity of morphological characters uniting Ecdysozoa, the group is named for the archetypal growth strategy that is shared among all its members; that is the periodical shedding of the cuticular exoskeleton in order to accommodate changes in body size and/or shape^5^. Ecdysis is intricately linked with ontogenetic development, and therefore most ecdysozoans will typically undergo several moulting cycles over their lifespan. This carries important implications for understanding the evolution of this process in deep time, as the fossil record provides insights on diverse moulting adaptations in extinct ecdysozoan groups^6^. All ecdysozoans have the potential to produce several exuviae (i.e. shed exoskeletons) throughout their lifetime, increasing the likelihood of becoming preserved in the rock record. However, most fossil evidence of moulting is biased in favour of organisms with heavily biomineralised exoskeletons (e.g. trilobites, decapod crustaceans)^7–10^, or those whose behaviour may lead to the burial of numerous exuviae simultaneously (e.g. eurypterid mass moulting events)^6,11^. By contrast, information on the moulting strategies of soft-bodied forms is comparatively rare, and only found under exceptional circumstances such as those conferred by Konservat-Lagerstätten^6,12–14^. Prominent instances of moulting in Cambrian soft-bodied forms include euarthropods (*Marrella*^12^, *Canadaspis*^15^, *Alacomenaeus*^13^, *Houlongdongella*^16^), lobopodians with stacked sclerites (*Onychodictyon*^17^, *Hallucigenia*^18,19^, *Collinsium*^20^, *Microdictyon*^21^), and scalidophoran worms (*Sirilorica*^14^, *Wronascolex*^22^). From these examples, however, only the crown-group euarthropod *Marrella splendens*^12^ and the stem-group loriciferan *Sirilorica pustulosa*^14^ capture the moment at which the old exoskeleton is discarded, and thus provide a narrow perspective on the diversity of ecdysis for early Ecdysozoa. Here, we describe the moulting behaviour of the fuxianhuiid *Alacaris mirabilis* from the early Cambrian (Stage 3) Xiaoshiba biota in South China^23^. Our findings offer the first characterisation of ecdysis in an upper stem-group euarthropod^24^, and illuminate the ancestral moulting behaviour within this diverse animal group.

## Results

The dorsal exoskeletal configuration of *Alacaris mirabilis* (Chengjiangocarididae, Fuxianhuiida)^23^ consists of a semicircular anterior sclerite with stalked lateral eyes^25^ that is articulated dorsally with a heart-shaped head shield covering the anterior body region. As with other fuxianhuiids^26–28^, the ventral surface of the head of *A. mirabilis* features a pair of pre-oral, multisegmented antennae, followed by well-developed specialised post-antennal appendages (SPAs) in a para-oral position. The mouth is covered by a broad sclerotised hypostome that overlies the basal portions of the SPAs. The trunk of *A. mirabilis* consists of 13 trunk tergites; the five anterior-most tergites are greatly reduced relative to the maximum body width and are covered by the head shield in life position. The remaining tergites gradually taper in width towards the posterior. Ventrally the trunk bears more than a dozen pairs of biramous limbs, including a differentiated gnathobasic protopodite (expressed only on the first three sets of post-oral appendages), a multipodomerous endopod, and a flap-like oval exopod with short marginal setae. The conical telson has paired paddle-like tail flukes fringed with posterior-facing elongate setae.

**Figure 1:**
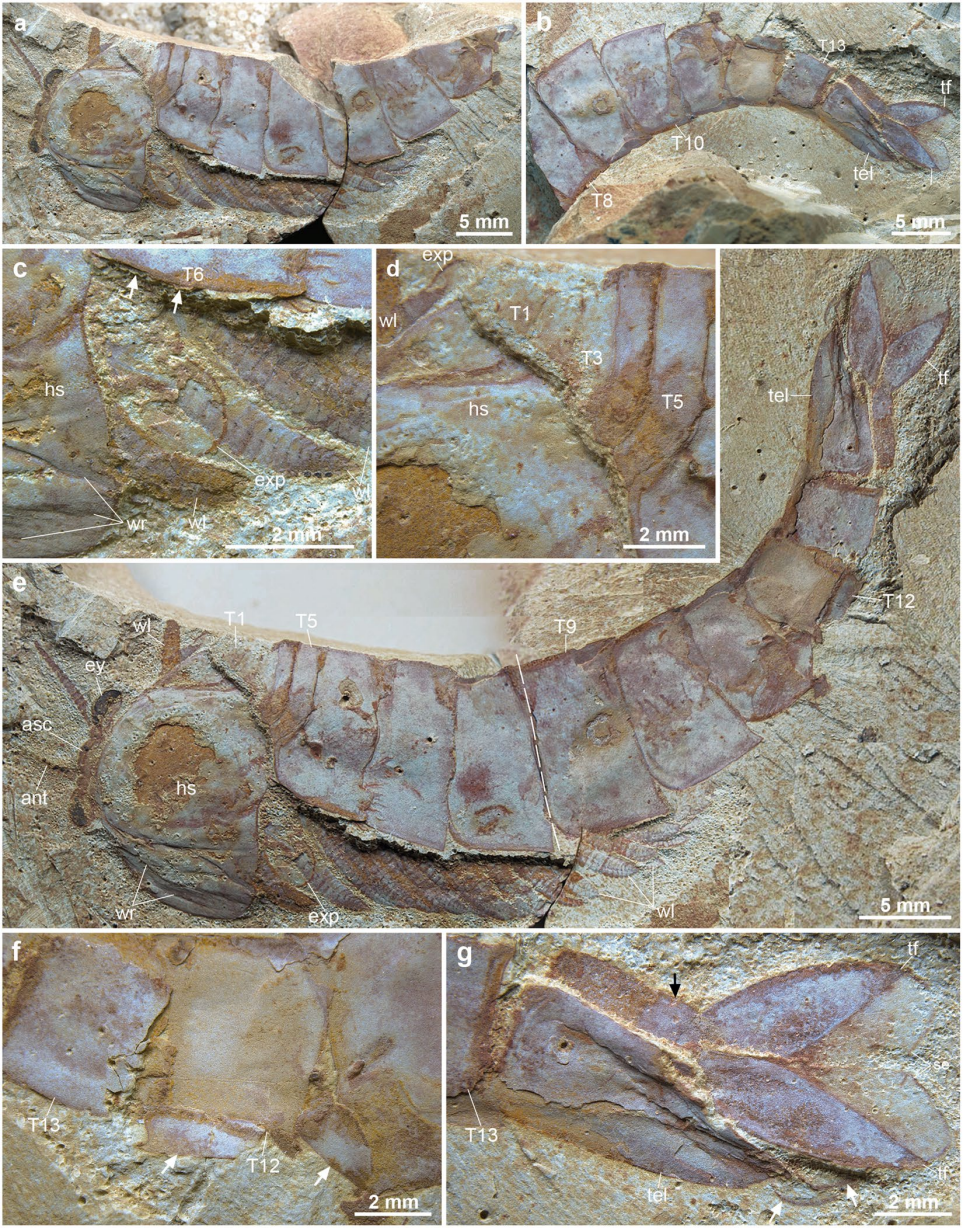
*Alacaris mirabilis* from the Cambrian (Stage 3) Xiaoshiba Lagerstätte in Kunming, southern China. (**a**) YKLP 12270a, dorsal view of the head shield and anterior portion of trunk. (**b**) YKLP 12270b (counterpart), showing the posterior portion of trunk and telson. (**c**) Close-up showing the layer of sediment (arrowed) separating the well sclerotized trunk tergites from the wrinkled head shield. (**d**) Close-up of the five reduced anterior tergites articulated to the remainder of the trunk, and overlapping the carcass head shield. (**e**) Restored specimen by digitally combining YKLP 12270a and 12270b by computer amalgamation (using photomerge software), the dashed line indicates the contact of YKLP 12270a by 12270b. (**f**) Close-up view of 11^th^ to 13^th^tergites with fragments of duplicated tergites (arrows). (**g**) Close-up of the moulted telson articulated with the complete trunk tergite series and displaying paired tail flukes (white arrows), overlapping the pristine underlying telson (black arrow) with complete flukes and posteriorly-pointing setae. Abbreviations: ant, antenna; asc, anterior sclerite; exp, exopod; ey, eye; hs, head shield; tel, telson; tf, tail fluke, T*n*, trunk tergites; wl, walking leg; wr, wrinkles.

**Figure 2:**
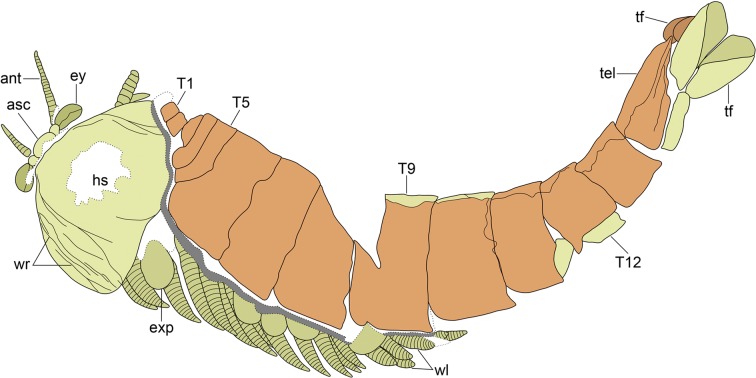
Interpretative diagram of YKLP 12270. Diagram based on the digitally reconstructed complete specimen depicted in Fig. 1e. The exuvia includes the entire trunk tergite series and telson (*orange*), and is preserved above the soft emerging individual represented by the head shield with articulated anterior sclerite, stalked eyes and antennae, as well as ventral limbs and duplicated telson with attached tail flukes (*yellow*). Abbreviations as in Fig. 1.

**Figure 3:**
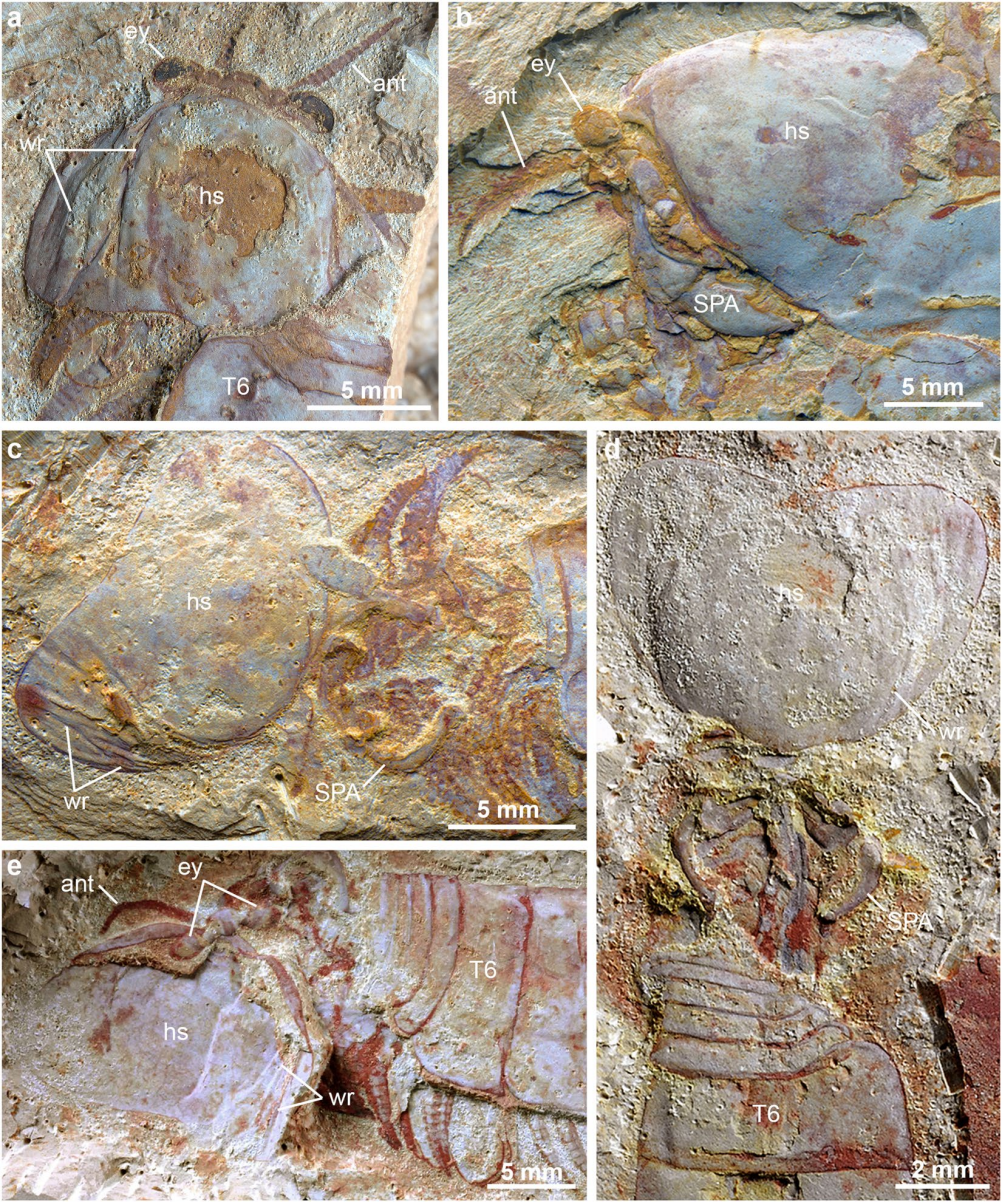
Taphonomic variability in specimens of *Alacaris mirabilis* and *Chengjiangocaris kunmingensis* from the early Cambrian Xiaoshiba biota. (**a**) *Alacaris mirabilis*, YKLP 12270, individual preserved in the act of moulting with wrinkled head shield, eyes and antennae in life position. (**b**) *A. mirabilis*, YKLP 12272, specimen preserved in life position. The presence of articulated eyes and antennae with the head shield, and absence of cuticular wrinkles, indicate that this specimen is a carcass from an inter-moult individual. (**c**) *A. mirabilis*, YKLP 12276, specimen preserved with ‘taphonomic dissection’, defined by the disarticulation of the head shield whilst still partially attached to the body anteriorly; note the presence of wrinkles on the head shield resulting from post-mortem compaction and decay. (**d**) *Chengjiangocaris kunmingensis*, YKLP 12020. (**e**) *C. kunmingensis*, YKLP 12024, specimen preserved with ‘taphonomic dissection’. Abbreviations: SPA, specialised post-antennal appendages; others as in Fig. 1.

Specimen YKLP12270 is an articulated dorso-ventrally flattened individual with well-preserved soft tissues, including the stalked eyes connected to the anterior sclerite, multisegmented antennae, and 19 sets of biramous trunk appendages (Figs 1 and 2). YKLP12270a preserves the anterior half of the individual (Fig. 1a). The counterpart YKLP12270b contains the posterior body termination (Fig. 1b); a digital composition of the part and counterpart photographs provides a clear view of the whole individual (Fig. 1e). The orientation of the specimen reveals most of the head shield and the entire left side of the trunk, including its corresponding limbs. The anterior right half of the trunk, comprising part of the first to eighth tergites, is missing. YKLP12270 is identified as *A. mirabilis* based on the possession of 13 trunk tergites, of which the five anterior-most are greatly reduced relative to the maximum body width (Fig. 1a,d). However, the configuration of the dorsal exoskeleton preserved in YKLP12270 differs substantially from that of the type material^23^, as well as other fuxianhuiids more generally^26,27,29,30^. The five reduced tergites of chengjiangocaridids are concealed beneath the head shield in life position^23,27,30^ (Fig. 3); by contrast, the entire trunk tergite sequence of YKLP 12270 is preserved on a level above that of the head shield and its associated structures (Figs 1a,d,e and 2), with the five reduced tergites overlapping the head shield but without indication of the trunk being displaced otherwise. The presence of a layer of sediment separating the head shield and biramous appendages from the overlying dorsal exoskeleton (Fig. 1c–e) demonstrates that these anatomical components were physically disconnected prior to burial.

A closer examination of individual structures reveals differences compared to previously described carcasses of *A. mirabilis*^23^ (Fig. 3b,c). For instance, the head shield of YKLP 12270 is connected with the eye-bearing anterior sclerite and the antennae, suggesting *in situ* burial with minimal taphonomic alteration due to transport or decay. However, the dorsal cuticle of the head shield also shows substantial lateral wrinkling and flattening (Figs 1a,e and 2). The texture of the head shield in YKLP 12270 (Fig. 3a) contrasts with the smooth surface typical of well-preserved chengjiangocaridids (Fig. 3b). Xiaoshiba fuxianhuiids frequently show the so-called “taphonomic dissection” of the head shield^27^, in which this structure is displaced forwards (and commonly overturned) but remains attached to the body through its connection close to the anterior sclerite (Fig. 3c–e). Fuxianhuiids with taphonomically-displaced head shields frequently have lateral wrinkles associated with softening of the cuticle, likely due to decay and post-burial compaction (Fig. 3c–e). The head shield of YKLP 12270 also shows wrinkling relative to the cuticle of the trunk tergites preserved in the same specimen, which have a smooth surface (Fig. 1a,b,e). In addition, the trunk tergites of YKLP 12270 show some irregularities. The distal pleural tips of the eleventh and twelfth tergites reveal aduplicated tergite underneath (Fig. 1b,f), clearly identifiable for the eleventh tergite because its lateral border is complete adjacent to this additional fragment. The presence of extra exoskeletal elements is also found in the posterior-most body region, as exemplified by the peculiar duplication of the telson (Figs 1b,e,g and 2). The upper telson articulates with the trunk tergites, possesses a slight three-dimensionality, has the typical conical morphology of *A. mirabilis*, and possesses two small, slightly displaced, flukes that likely correspond to the bases of the tail flukes (Figs 1g and 2). These two flukes are impressed with the left side, one on top of the other, which is consistent with the articulated trunk being preserved in a somewhat lateral orientation with only the left side fully displayed. The underlying complete telson is flattened and supports well-preserved tail flukes bearing delicate posterior-facing setae (Fig. 1g). Similar to the head shield and anterior trunk tergites, the duplicated telsons are separated by a layer of sediment. Lastly, the underlying telson and tail flukes have a dorsal orientation that parallels that of the head shield and appendages relative to the articulated trunk (Fig. 1b,e–g), and the overlying telson follows the curvature of the trunk.

**Figure 4:**
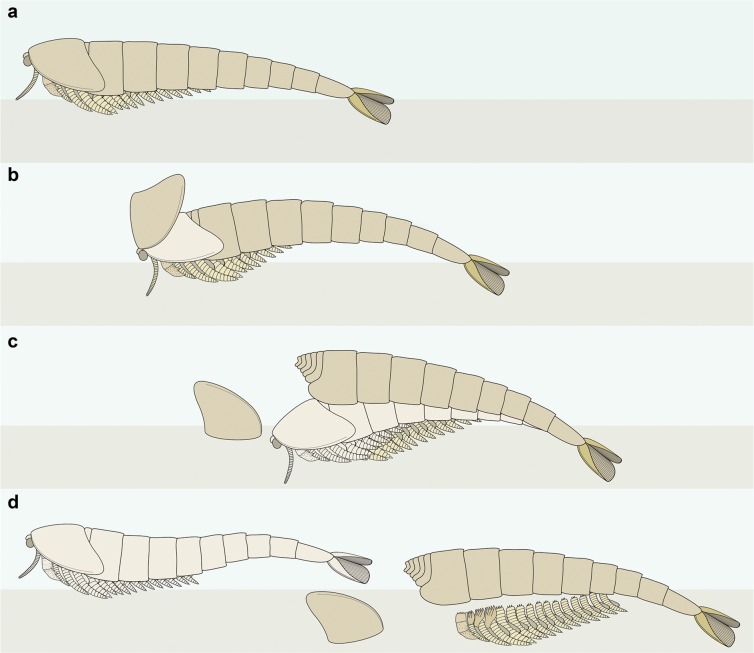
Reconstruction of ecdysis in *Alacaris mirabilis* based on the Open Moult Position strategy. (**a**) Inter-moult stage; note that the anterior reduced tergites are covered by the head shield in life position. (**b**) Ecdysis begins with the posterior detachment of the head shield, partially revealing the underlying anterior reduced tergites. (**c**) As the emerging individual pushes against the exuvia, the old head shield is inverted and separated from the body, and the appendages become separated from the trunk; the preservation of YKLP 12270 suggests it was buried during this stage. (**d**) The soft individual emerges completely through the resulting gape, leaving the partially articulated exuvia behind.

The preservation of YKLP 12270 differs from other fully articulated individuals of *A. mirabilis* from the Xiaoshiba biota, as well as other members of Chengjiangocarididae^22,26,29^ (Fig. 3). These taphonomic irregularities include: the overlap pattern of the dorsal trunk tergite series relative to the well-preserved head shield and associated *in situ* structures (Figs 1a,d and 2); wrinkling restricted to the head shield (Figs 1a,e and 3a); different orientations of the trunk series and head shield (Fig. 1a,b,e);and the duplication of tergite margins and the entire telson in the posterior body region (Fig. 1g). These taphonomic features suggest that YKLP 12270 represents an individual at the exuviation stage of moulting, buried during the process of shedding the old exoskeleton^31^. The well-sclerotised trunk tergite series with conical telson corresponds to the discarded exoskeleton, and lies above the actual carcass of the emerging individual (partially encased within the exuvia), represented by the head shield with articulated eyes and antennae, the sets of ventral biramous appendages, and the pristine telson with tail flukes (Fig. 2). This configuration implies that the old head shield corresponding to the exuvia is no longer present, having been separated from the body following disarticulation for moulting (Fig. 4b), and only the attached head shield of the carcass is visible on the specimen. Most of the dorsal trunk tergite series of the emerging individual – except for the posterior body termination – cannot be observed on the specimen. The missing trunk tergites are likely concealed within the layer of sediment separating the carcass from the discarded exoskeleton, and also obscured by the overlaying old tergite series (Fig. 1c–e). Based on the orientation of YKLP 12270, the moulting process of *A. mirabilis* most likely involved the posterior separation of the head shield from the body, comparable to the configuration observed in taphonomically-dissected fuxianhuiid specimens from the Xiaoshiba Lagerstätte^23,27^. This disarticulation pattern would facilitate the egress of the emerging individual through the resulting ample gape at the anterior of the body (Fig. 4d). The preservation of duplicated exoskeletal elements at the posterior of YKLP 12270b would have resulted from the lateral rupturing of the old exoskeleton during exuviation, revealing the new telson with articulated flukes and some tergite margins (Fig. 1b,e–g).

## Discussion

Comparison with the fossil record of euarthropod ecdysis^6,10^ further supports the interpretation of YKLP 12270 as an individual preserved while moulting. The lateral wrinkling of the flattened head shield closely evokes the appearance of rare trilobites that were buried shortly after moulting, and prior to the expansion and hardening of the new exoskeleton (i.e. the ‘soft shell’ stage)^6,9,31,32^. By comparison, the articulated trunk tergite series of YKLP 12270 completely lacks wrinkles (Fig. 1a,b,d,e), which is consistent with the typical well-sclerotised exoskeleton observed between moulting events (Fig. 3b). In soft-shelled trilobites the wrinkling is often confined to the lateral sections (Harriet Drage personal observation), similar to the situation observed in YKLP 12270 (Fig. 3a). The only other specimens of *A. mirabilis* that display a comparable degree of head shield wrinkling are those with taphonomically displaced head shields^23^ (Fig. 3c), which can be attributed to the effect of post-mortem decay and burial.

The overall configuration of the exoskeleton in YKLP 12270 broadly resembles that of some decapod crustacean fossils, particularly those of lobsters and shrimps. Decapod moults are readily recognisable in both extant and extinct representatives owing to their heavy exoskeleton calcification and correspondingly high preservation potential^6^. Decapod exuviae are often found in the so-called ‘Open Moult Position’, in which the posterior margin of the dorsal carapace (comparable to the head shield) becomes separated from the succeeding tergites and rests at a right angle relative to the trunk; the carapace often becomes displaced when the individual escapes through the resultant opening^33–35^ (Fig. 3). Fossilised decapod moults usually consist of the articulated trunk tergite series and a disarticulated carapace that often remains partly attached to the rest of the body^6^. Fossilised lobster moults may also show the similar ‘Overturned-Carapace Moult Position’, in which the carapace has entirely separated from the body and become inverted^34^, closely resembling the head shield configuration in taphonomically-dissected Xiaoshiba fuxianhuiids^23,27^ (Figs 3c,d and 4c,d). Despite the considerable phylogenetic distance between decapods and fuxianhuiids, the common moulting strategy of these organisms appears to be defined by the presence of a broad head shield or carapace covering the dorsal anterior body that needs to become disarticulated in order to enable exuviation. Due to the absence of the discarded head shield in YKLP 12270, it is not possible to unequivocally conclude that the head shield disarticulated at the posterior joint with the trunk as occurs in decapods. An alternative possibility could be an anterior disarticulation of the head shield and forwards exuviation as observed in some trilobites (e.g. harpetids and trinucleids)^31^ and aquatic chelicerates (e.g. eurypterids)^6^. However, we regard this option as less likely given the consistent articulation of the head shield to the anterior sclerite observed in taphonomically dissected fuxianhuiids^23,27^ (Fig. 3c–e), which suggests a strong attachment site that remained stable even after death, and not a point of weakness that would disarticulate with ease.

We consider that the moulting behaviour reconstructed for *A. mirabilis* was likely common among fuxianhuiids given their similar exoskeletal construction^23,27–30^, and potentially ancestral for members of Deuteropoda^24^ more generally. A decapod-like moulting style and production of the Open Moult Position is supported by instances of Cambrian bivalved euarthropods in which the carapace is preserved at a right angle relative to the trunk (see Fig. 8f in ref.^36^), and mass moulting assemblages of the megacheiran *Alalcomenaeus* sp. in which the head shield is consistently missing from the exuviae^13^. The Open Moult Position would appear to represent the common mechanism for performing ecdysis without the aid of specialized exoskeletal adaptations (e.g. suture lines), which are common in more phylogenetically-derived forms such as trilobites^7–9^, and appear to may have evolved multiple times amongst various groups of Cambrian euarthropods^37,38^. The occurrence of the Open Moult Position seems constrained by the overall construction of the exoskeleton, particularly in taxa characterized by the presence of an extensive head shield or carapace covering the anterior region. By contrast, benthic euarthropods that have a dorsoventrally flattened profile, or that possess a proportionally smaller head shield, usually perform ecdysis facilitated through the integration of weakness lines in the exoskeleton, coupled with different patterns of breakage of the exuviae^6–9,31^. Although the exoskeletal configuration of the fuxianhuiid head shield most closely resembles that of decapod crustaceans among extant representatives in this context, it does not represent a perfect comparison given differences in terms of the site of attachment of this structure. Whereas the carapace in decapods and other crustaceans originates from the dorsal side of the trunk^39,40^, the fuxianhuiid head shield is attached to the anterior margin of the head, and thus the similarities in mechanics of exuviation are most likely a result of convergent evolution resulting from a superficially similar exoskeletal organization.

**Figure 5:**
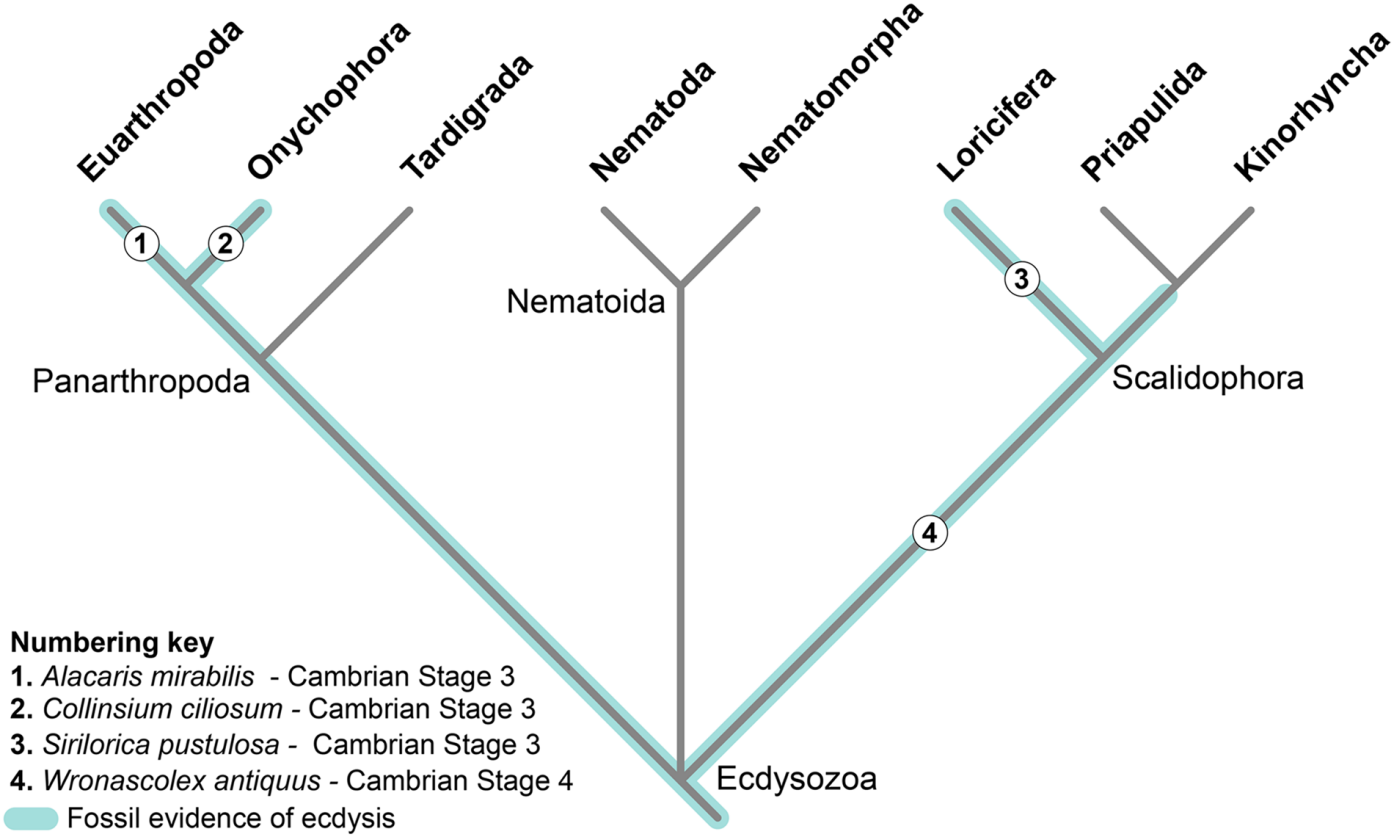
Simplified phylogeny of Ecdysozoa. Tree depicts the stratigraphically oldest and phylogenetically most ancestral fossil evidence of ecdysis for the major groups. Coloured lines indicate phylogenetic coverage of *Alacaris mirabilis*^23^, *Collinsium ciliosum*^20^, *Sirilorica pustulosa*^14^, and *Wronascolex antiquus*^22^. Topology based on refs^3,4^.

Specimen YKLP 12270 makes *A. mirabilis* the second Cambrian euarthropod known to date to have been preserved during ecdysis, and also the phylogenetically earliest diverging taxon that illustrates the moulting behaviour of total-group Euarthropoda (Fig. 5). The direct record of ecdysis in Cambrian euarthropods is extremely limited as freshly-moulted individuals^9,31,32^ are particularly vulnerable, and therefore cuticular hardening occurs as quickly as possible in order to avoid predation^6,41^. The other known case of a Cambrian euarthropod preserved whilst shedding the old exoskeleton is a specimen of *Marrella splendens* from the middle Cambrian (Wuliuan) Burgess Shale, whose soft emerging lateral spines are still partially trapped within the exuvia^12^. A stratigraphically-younger report of an individual of the Late Devonian trilobite *Trimerocephalus chopini* was described as preserved immediately post-exuviation^42^, but the original interpretation of this specimen has been questioned and is instead regarded as two overlapping carcasses^7^. *A. mirabilis* represents one of the stratigraphically-oldest ecdysozoan fossils that capture the process of moulting^6^ (Fig. 5). The only other specimen of comparable age that demonstrates ecdysis corresponds to the stem-group loriciferan *Sirilorica pustuolosa* from the Cambrian (Stage 3) Sirius Passet in North Greenland, where a complete emerging individual is associated with the shed lorica (Fig. 7 in ref.^14^). A specimen of the palaeoscolecid *Wronascolex antiquus* from the Cambrian (Stage 4) Emu Bay Shale in Australia has also been tentatively regarded as a putative exuvia (Fig. 3d in ref.^22^), although the authors did not rule out the possibility that it could also represent a decayed carcass. Despite the availability of additional Cambrian fossils that exemplify different modes of moulting^6^, none of them capture the moment of ecdysis. For example, armoured lobopodians possess dorsal hardened sclerites for protection located above most of the limb pairs throughout the trunk^43^. Data from complete body fossils and/or isolated sclerites indicate that these structures were replaced regularly, as shown by the presence of multiple stacked sclerites in the plates of *Onychodictyon*^17^, as well as the spines of *Hallucigenia*^18^, *Microdictyon*^21^, and the luolishaniid *Collinsium*^20^. Likewise, the terminal claws of *Hallucigenia* also possess multiple stacked elements, similar to the condition observed in extant onychophorans^19^. The rarity of direct fossil evidence demonstrating ecdysis in extinct organisms highlights the significance of the *A. mirabilis* specimen caught in the act of moulting. Our findings contribute towards a better understanding of the diversity and evolution of this critical growth strategy, which typifies the body plan of one of the most diverse and ecologically dominant group of animals that originated during the Cambrian Explosion.

## Methods

### Fossil repository and imaging

The study is based on a single specimen (YKLP 12270a,b) of the fuxianhuiid *Alacaris mirabilis*, Yang *et al*.^20^, from the Cambrian (Stage 3; local lower Canglangpuan Stage) Xiaoshiba section in Kunming, South China (see locality details in refs^20,27,37^). The specimen is deposited at the Key Laboratory for Palaeobiology, Yunnan University, Kunming, China. Fossils were photographed with a Nikon D3X fitted with a Nikon AF-S Micro Nikkor 105 mm lens, with directional illumination provided by a LEICA LED5000 MCITM.

## References

[1] Aguinaldo AMA (1997). Evidence for a clade of nematodes, arthropods and other moulting animals. Nature.

[2] Edgecombe GD (2009). Palaeontological and molecular evidence linking arthropods, onychophorans, and other Ecdysozoa. Evolution: Education and Outreach.

[3] Rota-Stabelli O, Daley AC, Pisani D (2013). Molecular timetrees reveal a Cambrian colonization of land and a new scenario for ecdysozoan evolution. Curr.Biol..

[4] Telford MJ, Bourlat SJ, Economou A, Papillon D, Rota-Stabelli O (2008). The evolution of the Ecdysozoa. Phil. Trans. R. Soc. Lond. B.

[5] Ewer J (2005). How the ecdysozoan changed its coat. PLoS Biol..

[6] Daley AC, Drage HB (2016). The fossil record of ecdysis, and trends in the moulting behaviour of trilobites. Arthropod Struct. Dev..

[7] Drage HB, Daley AC (2016). Recognising moulting behaviour in trilobites by examining morphology, development and preservation: Comment on Błażejowski *et al*. 2015. BioEssays.

[8] Drage HB, Holmes JD, García‐Bellido DC, Daley AC (2018). An exceptional record of Cambrian trilobite moulting behaviour preserved in the Emu Bay Shale, South Australia. Lethaia.

[9] Whittington HB (1990). Articulation and exuviation in Cambrian trilobites. Phil. Trans. R. Soc. Lond. B.

[10] Brandt S (2002). Ecdysial efficiency and evolutionary efficacy among marine arthropods: implications for trilobite survivorship. Alcheringa.

[11] Tetlie OE, Brandt DS, Briggs DE (2008). Ecdysis in sea scorpions (Chelicerata: Eurypterida). Palaeogeogr., Palaeoclimatol., Palaeoecol..

[12] García-Bellido DC, Collins DH (2004). Moulting arthropod caught in the act. Nature.

[13] Haug JT, Caron J-B, Haug C (2013). Demecology in the Cambrian: synchronized molting in arthropods from the Burgess Shale. BMC Biol..

[14] Peel JS, Stein M, Kristensen RM (2013). Life cycle and morphology of a Cambrian stem-lineage loriciferan. PloS One.

[15] Briggs DEG (1978). The morphology, mode of life, and affinities of *Canadaspis perfecta* (Crustacea: Phyllocarida), middle Cambrian, Burgess Shale, British Columbia. Phil. Trans. R. Soc. Lond. B.

[16] Zhang X-G (1987). Moult stages and dimorphism of Early Cambrian bradoriids from Xichuan, Henan, China. Alcheringa.

[17] Topper TP, Skovsted CB, Peel JS, Harper DA (2013). Moulting in the lobopodian Onychodictyon from the lower Cambrian of Greenland. Lethaia.

[18] Caron J-B, Smith MR, Harvey TH (2013). Beyond the Burgess Shale: Cambrian microfossils track the rise and fall of hallucigeniid lobopodians. Proc. R.Soc. Lond. B.

[19] Smith MR, Ortega-Hernández J (2014). *Hallucigenia*’ sonychophoran-like claws and the case for Tactopoda. Nature.

[20] Yang J (2015). A superarmored lobopodian from the Cambrian of China and early disparity in the evolution of Onychophora. Proc. Natl. Acad. Sci., USA.

[21] Chen JY, Zhou GQ, Ramsköld L (1995). The Cambrian lobopodian Microdictyon sinicum. Bull. Nat. Mus. Nat. Sci..

[22] García-Bellido DC, Paterson JR, Edgecombe GD (2013). Cambrian palaeoscolecids (Cycloneuralia) from Gondwana and reappraisal of species assigned to Palaeoscolex. Gondwana Res..

[23] Yang J (2018). Early Cambrian fuxianhuiids from China reveal origin of the gnathobasicprotopodite in euarthropods. Nat. Commun..

[24] Ortega‐Hernández J (2016). Making sense of ‘lower’ and ‘upper’stem‐group Euarthropoda, with comments on the strict use of the name Arthropoda von Siebold, 1848. Biol. Rev..

[25] Ortega-Hernández J (2015). Homology of head sclerites in Burgess Shale euarthropods. Curr. Biol..

[26] Chen J-Y, Zhou G-Q, Edgecombe GD, Ramsköld L (1995). Head segmentation in Early Cambrian Fuxianhuia: implications for arthropod evolution. Science.

[27] Yang J, Ortega-Hernández J, Butterfield NJ, Zhang X-G (2013). Specialized appendages in fuxianhuiids and the head organization of early euarthropods. Nature.

[28] Ortega-Hernández J, Yang J, Zhang X-G (2018). Fuxianhuiids. Curr. Biol..

[29] Waloszek D, Chen J-Y, Maas A, Wang X-Q (2005). Early Cambrian arthropods—new insights into arthropod head and structural evolution. Arthropod Struct. Dev..

[30] Hou X-G, Bergström J (1997). Arthropods of the lower Cambrian Chengjiang fauna, southwest China. Fossils Strata.

[31] Henningsmoen G (1975). Moulting in trilobites. Fossils Strata.

[32] Speyer SE, Brett CE (1985). Clustered trilobite assemblages in the Middle Devonian Hamilton group. Lethaia.

[33] Glaessner, M. F. Decapoda in *Treatise on Invertebrate Paleontology, Pt. R, Arthropoda* (ed. Moore, R. C.) 400–533 (Geol. Soc. Am. & Univ. Kansas Press, 1969).

[34] Bishop GA (1986). Taphonomy of the North American Decapods. Crust. Biol..

[35] Feldmann RM, Vega FJ, García-Barrera P, Rico-Montiel R, Martínez Lopez L (1995). A new species of Meyeria (Decapoda: Mecochiridae) from the San Juan Raya Formation (Aptian: Cretaceous), Puebla State, Mexico. J. Paleontol..

[36] Legg DA, Caron J-B (2014). New Middle Cambrian bivalved arthropods from the Burgess Shale (British Columbia, Canada). Palaeontology.

[37] Du, K. -S., Ortega-Hernández, J., Yang, J. & Zhang. X. -G. A soft-bodied euarthropod from the early Cambrian Xiaoshiba Lagerstätte of China supports a new clade of basal artiopodans with dorsal ecdysial sutures. *Cladistics* early view online, 10.1111/cla.12344 (2018). 10.1111/cla.12344 34622993

[38] Hou X-G (2017). A new species of the artiopodan arthropod Acanthomeridion from the lower Cambrian Chengjiang Lagerstätte, China, and the phylogenetic significance of the genus. J. Syst. Palaeontol..

[39] Møller OS, Olesen J, Høeg JT (2003). SEM studies on the early larval development of Triopscancriformis (Bosc) (Crustacea: Branchiopoda, Notostraca). Acta. Zool..

[40] Nazari EM, Müller YMR, Ammar D (2000). Embryonic development of Palaemonetes argentinus Nobili, 1901 (Decapoda, Palaemonidae), reared in the laboratory. Crustaceana.

[41] Bicknell RD, Paterson JR (2018). Reappraising the early evidence of durophagy and drilling predation in the fossil record: implications for escalation and the Cambrian Explosion. Biol. Rev..

[42] Błażejowski B, Gieszcz P, Brett CE, Binkowski M (2015). A moment from before 365 Ma frozen in time and space. Sci. Rep..

[43] Ortega-Hernández J (2015). Lobopodians. Curr. Biol..

